# Effect of keratinase on ileal amino acid digestibility in five feedstuffs fed to growing pigs

**DOI:** 10.5713/ajas.17.0815

**Published:** 2018-05-31

**Authors:** Chengfei Huang, Dongli Ma, Jianjun Zang, Bo Zhang, Brian Sun, Ling Liu, Shuai Zhang

**Affiliations:** 1 State Key Laboratory of Animal Nutrition, Ministry of Agriculture Feed Industry Centre, China Agricultural University, Beijing 100193, China; 2 Novus International, Inc, Shang Hai 200131, China

**Keywords:** Amino Acid, Apparent Ileal Digestibility, Feedstuff, Growing Pigs, Keratinase, Standardized Ileal Digestibility

## Abstract

**Objective:**

This study was conducted to evaluate the effect of keratinase (KE) on the apparent ileal digestibility (AID) and standardized ileal digestibility (SID) of amino acids (AA) in rice bran, cottonseed meal (CSM), rapeseed meal (RSM), corn distillers dried grains with solubles (DDGS), and peanut meal (PNM).

**Methods:**

Twelve crossbred barrows (Duroc×Landrace×Yorkshire, 50.5±1.4 kg body weight [BW]) fitted with T-cannulas at the terminal ileum were allotted to a 12×6 Youden Square design with 12 diets and 6 periods. The treatment diets included rice bran, CSM, RSM, corn DDGS, PNM, or corn-soybean meal (cSBM) supplemented with 0.05% KE or not. Diets were given to pigs at a level of 3% BW in two equal meals. The endogenous AA losses were the mean results of three previously experiments determined by a same nitrogen-free diet fed to pigs. Pigs had free access to water during the experiment.

**Results:**

The KE supplementation improved (p<0.05) the AID and SID of Met, Thr, Val, Asp, Cys, and Tyr in rice bran. Inclusion of KE increased (p<0.05) the AID and SID of Met and Val in CSM. The KE supplementation decreased (p<0.05) the AID and SID of His in RSM and all measured AA except for Arg, Met, Trp, Val, Gly, and Pro in corn DDGS. There was an increase (p<0.05) in AID and SID of Leu, Ile, Met, Ala, Cys, Ser, and Tyr in PNM supplemented with KE compared with that without KE. Inclusion of KE increased (p<0.05) the AID and SID of crude protein, Leu, Ile, Phe, Thr, Asp, and Ser in cSBM.

**Conclusion:**

This study indicated that KE had different effects on ileal AA digestibility of feedstuffs for growing pigs, which can give some usage directions of KE in swine feed containing those detected feedstuffs.

## INTRODUCTION

Keratinase (KE), one of the proteases, was first isolated from the culture medium of *Bacillus licheniformis PWD-1* [1,2]. Compared with most known proteases, KE displays a higher proteolytic activity [3] and can break down a wide range of proteins such as casein, collagen, elastin, and keratin as well as other proteins containing cysteine disulfide bonds [4–6]. Previous studies reported that dietary KE supplementation improved the nutrient digestibility and apparent ileal digestibility (AID) of amino acids (AA) for growing pigs [7]. It was also reported that KE could improve growth performance, breast meat yield, and gut villus structure of broilers fed diets based on corn and soybean meal (SBM) [8–10].

Soybean meal is the primary protein source of swine feed in many countries. Some soybean proteins, such as glycinin, β-conglycinin, trypsin inhibitors, lectins and other minor proteins, can negatively impact on intestinal morphology of pigs [11–13]. Cottonseed meal (CSM), rapeseed meal (RSM), peanut meal (PNM), rice bran, and corn distillers dried grains with solubles (DDGS) are common local by-products that can be used in pig feed in China. However, these feedstuffs are poorly digested by pig and the nutrient values are quite varied. Improving nutritional value of these feedstuffs for pigs, such as improving AA digestibility, becomes important when they are included as lower cost alternatives in pig diets. It is reasonable to hypothesize that KE can improve the AA digestibility of those feedstuffs, which may be rich in proteins containing cysteine disulfide bonds, according to the characteristics of KE. Therefore, the main objective of this study was to investigate the effects of KE on the ileal digestibility of crude protein (CP) and AA in five feedstuffs of rice bran, CSM, RSM, corn DDGS, and PNM for growing pigs. In addition, the effect of KE on the corn-soybean meal (cSBM) was also evaluated because the cSBM diet is commonly used in pig feed.

## MATERIALS AND METHODS

The trial protocol including animal care and use was approved by the Institutional Animal Care and Use Committee of China Agricultural University (Beijing, China).

### Preparation of keratinase

The KE was provided in the premix Cibenza DP100 (Novus International, Inc., Shang Hai, China, produced in 2015) and which was produced by *Bacillus licheniformis PWD-1*, after 48 h fermentation at 50°C, followed by concentrating and spray-drying [14]. The KE activity contained in this premix was more than 600,000 U/g where a unit is defined as an increase of 0.1 in absorbance at a wavelength of 280 nm under the conditions described by Gradišar et al [15].

### Animals and experimental design

This study was conducted to evaluate the AID and standardized ileal digestibility (SID) of CP and AA in five feed feedstuffs of rice bran, CSM, RSM, corn DDGS, and PNM in the Metabolism Laboratory of the Ministry of Agriculture Feed Industry Centre (China Agricultural University, Beijing, China). A cSBM diet that is commonly used in pig feed was also evaluated in the experiment. Twelve crossbred barrows (Duroc×Landrace ×Yorkshire, 50.5±1.4 kg body weight [BW]) fitted with T-cannula at the terminal ileum, using a method adapted from Stein et al [16], were allotted to a 12×6 Youden Square design with 6 periods and 12 diets. The experimental diets included rice bran, CSM, RSM, corn DDGS, PNM, or cSBM as the sole protein and AA source and which were each supplemented with 0.05% KE or not (Table 1). Chromic oxide was included at 0.3% as an indigestible index for calculating AA digestibility. The adjustment for basal endogenous CP and AA losses (g/kg dry matter intake) was based on estimates obtained from 3 previous studies conducted in our lab using pigs of similar BW and genetic background. The nitrogen-free diet in all 3 studies was a cornstarch-sugar-cellulose-soybean oil-based diet (73%, 15%, 4%, and 3% dietary inclusion, respectively). Values for endogenous CP and AA losses between the previous experiments were not different (i.e. p>0.11) thus a mean value was deemed suitable. The DM, CP, and AA composition of the experimental diets is presented in Table 2. The values of endogenous CP and AA losses are presented in Table 3. Pigs were weighed at the start of each experimental period and feed allotment adjusted accordingly. All pigs had free access to water during the experiment.

### Digesta collection and sampling

Each experimental period lasted for 7 d where the first 5 d were considered the adaptation period followed by 2 d of digesta collection. Digesta was collected continuously from 08:00 to 17:00 using procedures described by Stein et al [16]. Digesta was collected in plastic bags attached to the simple T-cannula. Bags were changed when digesta made up no more than 30% of the bag volume. Collected digesta was immediately stored in a −20°C freezer.

At the end of the experiment, ileal digesta was thawed and mixed within animal and diet, and a sub-sample of 500 mL was taken. Digesta samples were lyophilized in a vacuum-freeze dryer (Tofflon Freezing Drying Systems, Minhang District, Shanghai, China) and ground through a 1-mm screen for further chemical analysis.

### Chemical analyses

The DM, CP, and AA were analyzed in the feedstuffs, diets, and digesta. Feedstuffs were also analyzed for ether extract (EE), crude fiber (CF), neutral detergent fiber (NDF), acid detergent fiber (ADF) and ash. All experimental diets and digesta were analyzed for Cr to calculate the concentration of indigestible index chromic oxide. The DM, CP, and ash in samples were analyzed according to AOAC procedures (method 930.15, DM; method 984.13, CP; method 942.05, ash) [17] and EE was determined according to Thiex et al [18]. The CF, NDF, and ADF were determined using filter bags and fiber analyzer equipment (Fiber Analyzer, Ankom Technology, Macedon, NY, USA) following the procedures described by Van Soest et al [19]. The concentration of NDF was analyzed using heat stable α-amylase and sodium sulfite without correction for insoluble ash.

Determination of AA was conducted according to Li et al [20] where samples were hydrolyzed with 6 *N* HCl at 110°C for 24 h and then analyzed using an Amino Acid Analyzer (Hitachi L-8900, Tokyo, Japan). Sulfur-AA (Met and Cys) content of digesta, diets, and feedstuffs was determined using cold performic acid oxidation overnight and hydrolyzed with 7.5 *N* HCl at 110°C for 24 h before measurement using an Amino Acid Analyzer (Hitachi L-8900, Japan). Estimates of Trp were made by hydrolyzing samples with LiOH for 22 h at a constant temperature of 110°C and then analyzed using High Performance Liquid Chromatography (Agilent 1200 Series, Santa Clara, CA, USA). Analysis of the Cr concentration in all diets and digesta was conducted using a polarized Zeeman Atomic Absorption Spectrometer (Hitachi Z2000, Japan) after nitric acid-perchloric acid wet ash sample preparation. All chemical analyses were conducted in two duplicates. The analyzed composition of feedstuffs is presented in Table 4.

### Calculations

The AID of AA was calculated using the following method described by Stein et al [21]:

$$\text{AID}=[1-({\text{AA}}_{\text{d}}/{\text{AA}}_{\text{f}})\times ({\text{Cr}}_{\text{f}}/{\text{Cr}}_{\text{d}})]\times 100\%$$

Where, AA_d_ and Cr_d_ were the concentrations of AA and Cr in the ileal digesta (g/kg of DM) and AA_f_ and Cr_f_ were the concentrations of AA and Cr in the test diets (g/kg of DM). The SID of AA was calculated using the following equation:

$$\text{SID}=[\text{AID}+({\text{IAA}}_{\text{end}}/{\text{AA}}_{\text{f}})\times 100\%]$$

In which IAA_end_ is the basal endogenous loss of an AA (g/kg of DM intake). The AID and SID of CP were determined using the same two aforementioned equations.

### Statistical analysis

Data were checked for normality, and outliers were detected and then removed using the UNIVARIATE procedure of SAS (SAS Inst. Inc., Cary, NC, USA). Outliers were defined as those beyond the range of mean±3 times standard deviation. Data were then analyzed using MIXED procedure of SAS, and the statistical model included the fixed effects of feedstuff and KE supplementation, and their interaction effect, and the random effects of animal and period. Pig was treated as the experimental unit. Means were calculated using the LSMEANS statement, and multiple comparison were adjusted using Tukey’s test. Because the interaction effects between feedstuff and KE were significant for almost all the parameters we tested (AID and SID of AA), data were then analyzed within each feedstuff by one-way analysis of variance using the general linear model procedure of SAS to test the effects of KE within each ingredient. An α value of 0.05 was used to assess statistical significances among treatment means.

## RESULTS

The results of AID and SID of CP and AA are shown in Tables 5, 6, respectively. There was a significant interaction effect between feedstuff and KE supplementation for AID and SID of CP and almost all the AAs except for Arg, Ala, Gly, Pro, and Ser. Among the six tested diets, the cSBM diet and PM diet showed the greatest AID and SID for almost all the AAs, while rice bran diet and corn DDGS diet showed the lowest AID and SID for almost all the AAs.

Effects of KE on the AID of AA in feedstuffs analyzed within each diet in our study are shown in Table 7. Inclusion of KE improved (p<0.05) the AID of Met, Thr, Val, Asp, Cys, and Tyr in rice bran. The CSM supplemented with KE had greater AID of Met and Val compared with that in CSM without KE addition (p<0.05). The KE supplementation decreased (p<0.05) AID of His in RSM. The AID of all measured AA except for CP, Arg, Met, Trp, Val, Gly, and Pro in KE supplemented corn DDGS were lower (p<0.05) than that in corn DDGS without KE. Compared with PNM, the KE supplemented PNM had greater (p<0.05) AID of Leu, Ile, Met, Ala, Cys, Ser, and Tyr. The KE improved (p<0.05) the AID of CP, Leu, Ile, Phe, Thr, Asp, and Ser in cSBM.

Effects of KE on the SID of AA in feedstuffs analyzed within each diet in our study are shown in Table 8. Rice bran supplemented with KE had greater SID of Met, Thr, Val, Asp, Cys, and Tyr for pigs (p<0.05). The KE inclusion had greater (p< 0.05) SID of Met and Val in CSM. Similar to the effect on AID of AA, KE decreased (p<0.05) the SID of His in RSM. The SID of all measured AA except for Arg, Met, Trp, Val, Gly, and Pro were lower (p<0.05) in corn DDGS with KE supplementation than without. The SID of Leu, Ile, Met, Ala, Cys, Ser, and Tyr in PNM including KE were greater (p<0.05) than that in PNM without KE. The KE inclusion to the cSBM diet improved (p<0.05) the SID of CP, Leu, Ile, Phe, Thr, Asp, and Ser.

## DISCUSSION

In our study, the chemical composition of the rice bran was within the ranges reported by Shi et al [22]. The chemical composition of the CSM was within the ranges reported by Li et al [23]. The chemical composition of the RSM was within the ranges reported by Li et al [24]. The chemical composition of the corn DDGS was comparable to those values from NRC [25] (corn DDGS containing greater than 6% oil but less than 9% oil) and were within the ranges reported by Li et al [26]. The chemical composition of the PNM was comparable with the values reported by Li et al [27] and NRC [25]. The concentration of CP (47.46% as fed) in SBM was little higher than that of 45.1% reported by Lagos and Stein [28]. The concentration of individual AA in each tested feedstuff was within the relative standard deviation presented in NRC [25]. The values of endogenous CP and AA losses presented in Table 3 were comparable with the values from previous studies that also conducted in our lab [20,24,27].

The KEs are mainly serine and metalloproteases, which could hydrolyze protein without being denatured or broken down by the gastrointestinal tract in broiler chickens [9]. It may also need the presence of live cells for activating the sulfitolysis or reduction in disulfide bonds and proteolysis effect of KE [29,30]. Though the proteolysis effection of most proteases may begin when the animals consumed the feed, most digestive enzymes are not stable at the low pH encountered in the stomach and upper small intestine. As a result, these enzymes may become inactivated, or a portion of the enzyme is inactivated. This process of inactivation is the likely cause of the variable results of other enzymes reported previously. Microbial KEs are predominantly extracellular when grow on keratinous substrates and can be stable over a wide range of pH from 5 to 13 [31]. The optimal pH for KE produced by *Bacillus licheniformis PWD-1* was about 7.5 [2], which is like the conditions in the lower jejunum and ileum of pigs [32]. Therefore, the KE included in the current study was likely exerting its effect on protein digestion in the lower small intestine.

The significant interaction effect between feedstuff and KE supplementation indicated that the effect of KE on AA digestibility can be influenced by treatment diets. Considering the more abundant AA contents in cSBM diet and PM diet, and the relative poor AA contents in rice bran diet and corn DDGS diet, it is reasonable to observe the greatest AID and SID of almost all the AAs in cSBM and PM diets, while the lowest AID and SID of almost all the AAs in rice bran and corn DDGS diets. The improved AID of CP and AA in the KE supplemented cSBM diet agreed with Wang et al [7] where the same level of KE (0.05%) supplementation in a cSBM basal diet significantly increased the dietary AID of CP, Arg, His, Leu, Phe, and Thr. About 40% and 30% of the total soybean globulin proteins were glycinin and β-conglycinin, respectively [11,12]. The ratio of CP provided by SBM vs corn was 2.1:1 in cSBM diet used in our trial. Therefore, the improved digestibility of CP and AA in the KE added cSBM-based diet is most likely due to the digestion of glycinin and β-conglycinin, which were done via protein-disulfide reductase break down of the cystine disulfide bond and the peptidohydrolase hydrolyzation of the denatured protein into peptides and AA [31,33]. The KE has been used to improve digestibility of feather meal in livestock diets [34] and in poultry diets [8,10,35]. However, no previous studies reported the utilization of KE on the other feed ingredients.

The KE significantly improved the AID and SID of Met in rice bran, CSM, and PNM but not in RSM and cSBM. We supposed that this is related to the relative lower concentration of Met in the former three feedstuffs, considering relative less disulfide bonds that KE may need to break down. In rice bran, both the AID and SID of Met and Cys improved more than 20%, which can also primarily attribute to break down effect of KE on disulfide bonds of Met and Cys. In CSM, KE only affected the AID and SID of Met and Val, but not the dispensable AA. In RSM, KE supplementation only improved the AID and SID of His. In addition to Met, KE also improved the AID and SID of branched AA of Leu and Ile and some dispensable AA in PNM.

Although KE supplementation significantly improved the AID and SID of some AA in the five detected feedstuffs, it only significantly increased the CP digestibility in cSBM. In the current study, the CP levels in the detected diets except for rice bran diet were similar to those reported by Wang et al [7], who also found that there was no interaction effect between the dietary KE (0.05% vs 0.10%) and CP (22.0% vs 20.0%). Therefore, the various CP levels may not explain the discrepancies of the KE supplementation effects on CP digestibility in different feedstuffs. Moreover, the relative lower digestibility of Lys in corn DDGS compared with that in the other feedstuffs was reasonable, because of the heat damage during fermentation or drying during the processing of DDGS [36, 37]. However, the reasons for the reduction of AID and SID of most AA in corn DDGS supplemented with KE were not known. This is a new finding that has never been reported before. The possible hypothesis may be that some components in corn DDGS may have negative effects on activity of KE. Future studies are needed to explore the underlying mechanisms.

## CONCLUSION

This study indicated that KE had different effects on the ileal AA digestibility of feedstuffs for growing pigs. In corn DDGS, KE reduced the ileal digestibility for almost all the AAs. These findings could give some directions for KE utilization in swine feed containing cSBM, corn DDGS, CSM, RSM, PNM, and rice bran.

## Figures and Tables

**Table 1:** Ingredient composition of the experimental diets (as-fed basis, %)

Ingredients	Rice bran diet	Rice bran diet+DP100	Cottonseed meal diet	Cottonseed meal diet+ DP1001)	Rapeseed meal diet	Rapeseed meal diet + DP100	Corn DDGS diet	Corn DDGS diet+DP100	Peanut meal diet	Peanut meal diet +DP100	Corn SBM diet	Corn SBM diet + DP100
Rice bran	69.97	69.95	-	-	-	-	-	-	-	-	-	-
Cottonseed meal	-	-	37.98	37.98	-	-	-	-	-	-	-	-
Canola meal	-	-	-	-	43.48	43.48	-	-	-	-	-	-
Corn DDGS	-	-	-	-	-	-	58.97	58.97	-	-	-	-
Peanut meal	-	-	-	-	-	-	-	-	34.98	34.98	-	-
Corn starch	15.73	15.75	37.72	37.72	32.22	32.22	26.73	26.73	45.72	45.72	-	-
Corn	-	-	-	-	-	-	-	-	-	-	69.97	69.97
Soybean meal	-	-	-	-	-	-	-	-	-	-	25.73	25.73
Sucrose	10.00	10.00	20.00	20.00	20.00	20.00	10.00	10.00	15.00	15.00	-	-
Dicalcium phosphate	3.00	3.00	3.00	3.00	3.00	3.00	3.00	3.00	3.00	3.00	3.00	3.00
Sodium chloride	0.45	0.45	0.45	0.45	0.45	0.45	0.45	0.45	0.45	0.45	0.45	0.45
Chromic oxide	0.30	0.30	0.30	0.30	0.30	0.30	0.30	0.30	0.30	0.30	0.30	0.30
DP100	-	0.05	-	0.05	-	0.05	-	0.05	-	0.05	-	0.05
Starch	0.05	0.00	0.05	0.00	0.05	0.00	0.05	0.00	0.05	0.00	0.05	0.00
Vit-min premix2)	0.50	0.50	0.50	0.50	0.50	0.50	0.50	0.50	0.50	0.50	0.50	0.50
Total	100.00	100.00	100.00	100.00	100.00	100.00	100.00	100.00	100.00	100.00	100.00	100.00

DDGS, distillers dried grains with solubles; SBM, soybean meal.

1) DP100: Keratinase, provided by Novus International, Inc.

2) Vit-min premix provided the following quantities of vitamins and microminerals per kilogram of the diet for pigs: vitamin A, 5,600 IU; vitamin D_3_, 2,200 IU; vitamin E, 21.6 IU; vitamin K_3_, 1.8 mg; vitamin B_12_, 12 μg; thiamine, 0.88 mg; riboflavin, 4 mg; pantothenic acid, 10 mg; niacin, 20 mg; choline chloride, 0.32 g; folacin, 0.4 mg; pyridoxine, 1.8 mg; biotin, 40 μg; Fe (FeSO_4_·H_2_O), 88 mg; Cu (CuSO_4_·5H_2_O), 120 mg; Zn (ZnO), 96 mg; Mn (MnO), 16 mg; I (KI), 0.24 mg; and Se (Na_2_SeO_3_), 0.4 mg.

**Table 2:** Analyzed composition of the experimental diets (as-fed basis, %)1)

Item	Rice bran diet	Rice bran diet+DP1002)	Cottonseed meal diet	Cottonseed meal diet +DP100	Rapeseed meal diet	Rapeseed meal diet	Corn DDGS diet	Corn DDGS diet+DP100	Peanut meal diet	Peanut meal diet +DP100	Corn SBM diet	Corn SBM diet +DP100
Dry matter	89.54	89.81	91.08	91.05	91.27	91.16	90.02	90.50	89.86	90.88	88.31	88.41
Crude protein	11.20	11.17	19.47	19.91	18.83	18.59	17.81	18.18	20.53	20.00	20.03	20.57
Indispensable amino acid												
Arg	0.77	0.74	2.17	2.12	1.02	0.96	0.70	0.72	1.98	2.17	1.19	1.10
His	0.32	0.31	0.53	0.57	0.56	0.43	0.52	0.44	0.48	0.55	0.56	0.55
Ile	0.84	0.81	1.21	1.19	1.50	1.37	2.50	2.44	1.39	1.52	1.98	2.01
Leu	0.34	0.33	0.60	0.58	0.80	0.67	0.60	0.58	0.60	0.72	0.78	0.82
Lys	0.54	0.52	0.74	0.69	0.98	0.93	0.38	0.32	0.54	0.58	0.94	0.98
Met	0.11	0.15	0.16	0.18	0.28	0.23	0.23	0.26	0.10	0.12	0.21	0.18
Phe	0.44	0.42	1.02	1.00	0.65	0.64	0.87	0.85	1.01	1.11	0.99	0.98
Thr	0.42	0.42	0.57	0.57	0.78	0.72	0.61	0.61	0.53	0.59	0.73	0.74
Trp	0.12	0.13	0.21	0.21	0.26	0.25	0.11	0.11	0.17	0.19	0.24	0.21
Val	0.50	0.51	0.77	0.89	1.09	0.91	0.84	0.82	0.79	0.95	1.03	1.05
Dispensable amino acid												
Ala	0.63	0.62	0.70	0.72	0.85	0.80	1.30	1.29	0.77	0.84	1.00	1.02
Asp	0.97	0.95	1.68	1.63	1.23	1.16	1.10	1.09	2.17	2.39	1.84	1.92
Cys	0.17	0.20	0.25	0.25	0.45	0.41	0.30	0.30	0.21	0.21	0.29	0.29
Glu	1.26	1.20	3.35	3.39	3.06	2.85	3.02	3.01	3.48	3.82	3.25	3.39
Gly	0.55	0.53	0.75	0.71	0.91	0.84	0.64	0.65	1.09	1.18	0.75	0.78
Pro	0.54	0.46	0.88	0.76	1.30	1.19	1.51	1.55	0.98	1.11	1.27	1.30
Ser	0.48	0.46	0.71	0.73	0.75	0.70	0.76	0.79	0.85	0.92	0.88	0.91
Tyr	0.21	0.21	0.49	0.36	0.32	0.27	0.43	0.41	0.37	0.60	0.31	0.32

DDGS, distillers dried grains with solubles; SBM, soybean meal.

1) Data were analyzed in duplicates.

2) DP100: Keratinase, provided by Novus International, Inc.

**Table 3:** Basal endogenous CP and AA losses based on estimates obtained from 3 previous studies (g/kg dry matter intake)1)

Items	Average	1st study	2nd study	3rd study	SEM	p-value
CP	8.73	8.61	9.06	8.62	1.42	0.97
Indispensable amino acid						
Arg	0.25	0.19	0.14	0.31	0.07	0.23
His	0.08	0.12	0.08	0.06	0.01	0.12
Ile	0.22	0.25	0.22	0.21	0.04	0.65
Leu	0.46	0.42	0.36	0.54	0.08	0.29
Lys	0.28	0.29	0.26	0.29	0.05	0.90
Met	0.07	0.11	0.09	0.10	0.03	0.81
Phe	0.41	0.30	0.45	0.46	0.07	0.23
Thr	0.35	0.36	0.33	0.35	0.06	0.93
Trp	0.08	0.08	0.10	0.08	0.01	0.45
Dispensable amino acid						
Ala	0.40	0.41	0.33	0.44	0.09	0.65
Asp	0.49	0.45	0.46	0.53	0.09	0.77
Cys	0.25	0.26	0.26	0.24	0.04	0.78
Glu	0.68	0.68	0.72	0.66	0.13	0.92
Gly	0.67	0.58	0.49	0.78	0.22	0.53
Pro	0.39	0.38	0.38	0.40	0.16	0.97
Ser	0.29	0.27	0.29	0.29	0.05	0.96
Tyr	0.14	0.12	0.15	0.10	0.04	0.86
Val	0.37	0.29	0.38	0.40	0.06	0.50

CP, crude protein; AA, amino acids; SEM, standard error of means.

1) The 3 studies were conducted in the same lab using pigs of similar body weight and genetic background (unpublished data). The nitrogen-free diet used in the 3 studies have same formulations. These data were comparable with the values from previous studies that also conducted in our lab [20,24,27].

**Table 4:** Analyzed composition of the ingredients used in the experiment (as-fed basis, %)1)

Items	Rice bran	Cottonseed meal	Rapeseed meal	Corn DDGS	Peanut meal	Corn	Soybean meal
Proximate composition							
DM	90.07	90.78	88.06	90.78	90.87	87.21	90.30
CP	13.42	50.76	38.75	27.61	51.54	8.26	47.46
EE	16.60	0.50	1.16	8.12	3.15	3.19	2.40
CF	9.63	14.08	10.53	8.77	5.12	2.46	5.71
NDF	21.45	26.70	24.49	31.47	16.76	12.95	11.99
ADF	9.39	17.70	17.04	13.50	8.35	2.84	6.45
Ash	8.70	6.29	6.61	4.28	6.12	1.13	6.25
Indispensable amino acid							
Arg	0.95	5.24	2.11	0.91	5.46	0.30	3.24
His	0.36	1.25	1.08	0.74	1.23	0.22	1.27
Ile	0.50	1.30	1.40	0.86	1.61	0.27	2.04
Leu	1.05	2.74	2.88	3.78	3.60	1.25	3.94
Lys	0.70	1.81	2.20	0.56	1.53	0.23	2.93
Met	0.18	0.41	0.55	0.42	0.30	0.12	0.41
Phe	0.57	2.34	1.32	1.26	2.60	0.39	2.41
Thr	0.51	1.40	1.59	0.92	1.36	0.28	1.84
Trp	0.14	0.53	0.55	0.16	0.48	0.06	0.59
Val	0.66	1.95	1.94	1.21	2.00	0.34	2.33
Dispensable amino acid							
Ala	0.76	1.64	1.61	1.85	1.96	0.56	1.98
Asp	1.21	4.02	2.53	1.61	5.83	0.53	5.29
Cys	0.26	0.66	0.88	0.51	0.57	0.15	0.59
Glu	1.57	8.01	5.97	4.42	9.02	1.40	7.88
Gly	0.69	1.73	1.83	0.94	2.93	0.29	1.96
Pro	0.69	1.85	2.45	2.21	2.35	0.79	2.50
Ser	0.55	1.71	1.46	1.12	2.15	0.35	2.21
Tyr	0.34	0.98	0.78	0.81	1.56	0.22	1.29

DDGS, distillers dried grains with solubles; DM, dry matter; EE, ether extract; CF, crude fiber; NDF, neutral detergent fiber; ADF, acid detergent fiber.

1) Data were analyzed in duplicates.

**Table 5:** Apparent ileal digestibility (AID) of crude protein (CP) and amino acids (AA) in 6 experimental diets with or without keratinase addition fed to pigs (%)1)

Items	CP	Arg	His	Ile	Leu	Lys	Met	Phe	Thr	Trp	Val	Ala	Asp	Cys	Glu	Gly	Pro	Ser	Tyr
Diets																			
Rice bran	53.9^d^	82.3^c^	68.3^c^	58.5^e^	66.0^e^	63.6^c^	62.9^d^	62.1^d^	54.2^c^	63.0^b^	56.2^d^	59.6^d^	59.2^c^	47.1^c^	69.8^d^	36.9^b^	50.3^b^	56.9^e^	59.9^d^
Cottonseed meal	73.9^b^	91.4^ab^	72.2^bc^	76.9^c^	78.2^d^	62.8^c^	69.8^c^	85.3^b^	68.2^b^	76.7^a^	78.6^b^	66.9^c^	80.6^a^	72.3^a^	86.7^ab^	63.4^a^	53.1^b^	75.6^bc^	78.9^b^
Rapeseed meal	70.1^b^	82.9^c^	77.6^b^	78.3^bc^	79.5^cd^	75.7^b^	87.0^b^	78.5^c^	69.3^b^	68.2^b^	78.6^b^	75.2^ab^	71.6^b^	77.5^a^	84.7^b^	65.8^a^	67.5^ab^	71.8^cd^	70.1^c^
Corn DDGS	61.3^c^	80.4^c^	70.4^c^	69.2^d^	81.9^c^	41.5^d^	87.0^b^	79.6^c^	57.5^c^	44.4^c^	65.8^c^	72.1^bc^	62.8^c^	66.5^b^	76.7^c^	44.0^b^	64.1^ab^	69.3^d^	83.1^ab^
Peanut meal	79.2^a^	92.9^a^	84.4^a^	87.7^a^	89.4^a^	75.1^b^	92.6^a^	92.1^a^	76.9^a^	81.7^a^	89.0^a^	79.6^a^	83.4^a^	76.9^a^	88.8^a^	61.1^a^	62.3^ab^	81.3^a^	87.4^a^
Corn SBM	79.1^a^	89.4^b^	84.2^a^	82.8^b^	85.5^b^	83.3^a^	90.4^ab^	85.7^b^	75.8^a^	81.2^a^	80.0^b^	79.8^a^	80.7^a^	75.4^a^	84.9^b^	68.6^a^	77.3^a^	80.5^ab^	82.1^ab^
SEM	1.0	0.7	1.4	1.1	0.9	1.2	1.0	0.9	1.2	1.5	1.1	1.4	1.1	1.3	0.9	2.0	4.6	1.3	1.4
Keratinase (KE)2)																			
−	69.1	86.5	77.0	75.0	79.6	67.6	79.8	79.9	65.9	69.4	74.0	71.2	72.2	68.6	81.2	56.1	63.9	71.9	76.1
+	70.1	86.6	75.4	76.1	80.5	66.4	83.5	81.3	68.0	69.0	75.5	73.2	74.0	69.9	82.6	57.1	60.7	73.3	77.8
SEM	0.6	0.4	0.8	0.6	0.5	0.7	0.6	0.5	0.7	0.9	0.6	0.8	0.7	0.8	0.5	1.2	3.2	0.7	0.8
p-Value																			
Diets	<0.01	<0.01	<0.01	<0.01	<0.01	<0.01	<0.01	<0.01	<0.01	<0.01	<0.01	<0.01	<0.01	<0.01	<0.01	<0.01	<0.01	<0.01	<0.01
KE	0.22	0.90	0.16	0.19	0.23	0.24	<0.01	0.055	0.039	0.72	0.10	0.085	0.056	0.23	0.058	0.55	0.49	0.17	0.14
Diets×KE	<0.01	0.057	<0.01	<0.01	0.023	<0.01	<0.01	0.026	0.019	<0.01	<0.01	0.059	0.013	<0.01	0.032	0.25	0.27	0.11	<0.01

DDGS, distillers dried grains with solubles; SBM, soybean meal; SEM, standard error of means.

1) Data of digestibility were mean of six replicates. Data within a column without common superscripts differ (p<0.05).

2) “−” represents diets without keratinase addition, and “+” represents diets with keratinase addition.

**Table 6:** Standardized ileal digestibility (SID) of crude protein (CP) and amino acids (AA) in 6 experimental diets with or without keratinase addition fed to pigs (%)1)

Items	CP	Arg	His	Ile	Leu	Lys	Met	Phe	Thr	Trp	Val	Ala	Asp	Cys	Glu	Gly	Pro	Ser	Tyr
Diets																			
Rice bran	54.7^d^	82.7^c^	68.6^c^	59.2^e^	66.6^e^	64.1^c^	63.7^d^	63.0^d^	55.0^c^	63.7^b^	57.0^d^	60.3^d^	59.7^c^	48.5^c^	70.3^d^	38.1^b^	52.2^b^	57.5^e^	60.6^d^
Cottonseed meal	74.3^b^	91.5^ab^	72.4^bc^	77.2^c^	78.4^d^	63.2^c^	70.4^c^	85.7^b^	68.8^b^	77.1^a^	79.1^b^	67.5^c^	80.9^a^	73.3^a^	86.9^ab^	64.2^a^	54.1^b^	76.0^bc^	79.3^b^
Rapeseed meal	70.6^b^	83.2^c^	77.8^b^	78.6^bc^	79.8^cd^	76.0^b^	87.4^b^	79.2^c^	69.8^b^	68.5^b^	79.0^b^	75.7^ab^	72.0^b^	78.1^a^	84.9^b^	66.6^a^	68.0^ab^	72.2^cd^	70.6^c^
Corn DDGS	61.8^c^	80.7^c^	70.6^c^	69.6^d^	82.0^c^	42.3^d^	87.4^b^	80.1^c^	58.1^c^	45.1^c^	66.2^c^	72.4^bc^	63.3^c^	67.3^b^	76.9^c^	44.7^b^	64.6^ab^	69.7^d^	83.5^ab^
Peanut meal	79.6^a^	93.1^a^	84.6^a^	88.0^a^	89.8^a^	75.6^b^	93.5^a^	92.5^a^	77.5^a^	82.1^a^	89.5^a^	80.2^a^	83.6^a^	78.1^a^	88.9^a^	61.7^a^	62.4^ab^	81.7^a^	87.9^a^
Corn SBM	79.5^a^	89.6^b^	84.3^a^	83.1^b^	85.8^b^	83.6^a^	90.9^ab^	86.1^b^	76.2^a^	81.6^a^	80.3^b^	80.2^a^	81.0^a^	76.3^a^	85.1^b^	69.5^a^	78.0^a^	80.8^ab^	82.6^ab^
SEM	1.0	0.7	1.4	1.1	0.9	1.2	1.0	0.9	1.2	1.5	1.1	1.4	1.1	1.3	0.9	2.0	4.6	1.3	1.4
Keratinase (KE)2)																			
−	69.6	86.8	77.2	75.4	80.0	68.0	80.4	80.4	66.5	69.9	74.4	71.7	72.5	69.7	81.5	57.0	64.7	72.3	76.5
+	70.6	86.8	75.5	76.5	80.8	66.9	84.1	81.8	68.6	69.5	75.9	73.7	74.3	70.9	82.9	58.0	61.6	73.7	78.3
SEM	0.6	0.4	0.8	0.6	0.5	0.7	0.6	0.5	0.7	0.9	0.6	0.8	0.7	0.8	0.5	1.2	3.2	0.7	0.8
p-value																			
Diets	<0.01	<0.01	<0.01	<0.01	<0.01	<0.01	<0.01	<0.01	<0.01	<0.01	<0.01	<0.01	<0.01	<0.01	<0.01	<0.01	<0.01	<0.01	<0.01
KE	0.22	0.89	0.17	0.19	0.22	0.25	<0.01	0.054	0.040	0.72	0.10	0.086	0.055	0.24	0.058	0.54	0.50	0.17	0.14
Diets×KE	<0.01	0.055	<0.01	<0.01	0.023	<0.01	<0.01	0.026	0.020	<0.01	<0.01	0.059	0.013	<0.01	0.032	0.24	0.26	0.11	<0.01

DDGS, distillers dried grains with solubles; SBM, soybean meal; SEM, standard error of means.

1) Data of digestibility were mean of six replicates. Data within a column without common superscripts differ (p<0.05).

2) “−” represents diets without keratinase addition, and “+” represents diets with keratinase addition.

**Table 7:** Apparent ileal digestibility (AID) of crude protein (CP) and amino acids (AA) in 6 experimental diets with or without keratinase addition fed to pigs analyzed within each diet (%)1)

Items	−2)	+2)	SEM	p-value	−	+	SEM	p-value	−	+	SEM	p-value
		
	Rice bran diet				Cottonseed meal diet				Rapeseed meal diet			
CP	51.7	56.0	1.5	0.066	71.8	75.9	1.4	0.059	68.8	71.4	1.3	0.18
Indispensable AA												
Arg	82.0	82.7	0.7	0.47	90.8	92.0	0.8	0.30	81.8	84.1	1.2	0.20
His	70.5	66.2	2.4	0.23	70.8	73.7	3.3	0.55	79.6	75.6	1.0	0.022
Leu	64.6	67.5	1.0	0.097	76.2	79.9	2.0	0.22	79.9	79.1	1.6	0.75
Ile	57.9	60.9	1.3	0.13	74.8	78.9	2.2	0.20	79.4	77.2	1.8	0.41
Lys	62.0	65.2	1.6	0.18	60.5	65.0	2.4	0.21	75.5	75.9	1.2	0.81
Met	54.1	71.7	2.1	<0.01	68.5	71.1	0.8	0.040	89.5	84.6	1.7	0.062
Phe	59.8	64.3	1.6	0.073	84.0	86.6	1.5	0.24	77.6	79.4	1.7	0.48
Thr	51.7	56.6	1.5	0.035	65.4	71.0	2.7	0.17	68.8	69.8	2.2	0.74
Trp	61.6	64.4	1.7	0.27	74.7	78.8	1.3	0.056	70.88	65.6	3.2	0.28
Val	54.6	59.2	1.0	<0.01	74.6	82.6	1.9	<0.05	80.7	76.6	2.0	0.18
Dispensable AA												
Ala	59.1	61.2	0.9	0.11	63.3	70.5	2.9	0.10	73.8	73.5	1.3	0.85
Asp	56.9	61.6	1.1	0.012	78.9	82.4	1.9	0.20	69.8	73.4	2.8	0.38
Cys	41.9	52.3	2.9	0.027	71.7	72.9	2.1	0.67	77.2	77.7	1.0	0.74
Glu	70.0	70.9	0.8	0.43	84.9	88.4	1.2	0.068	83.8	85.5	1.8	0.52
Gly	35.1	38.6	3.5	0.49	62.7	63.9	2.9	0.77	62.6	68.9	2.5	0.10
Pro	49.8	51.3	3.6	0.83	53.1	53.2	7.0	0.99	65.1	70.0	7.4	0.68
Ser	55.1	58.7	1.4	0.099	73.1	78.0	2.2	0.15	70.9	72.7	2.3	0.60
Tyr	54.6	65.1	3.3	0.042	79.6	78.2	2.2	0.67	70.4	69.7	1.9	0.81
			
	**Corn DDGS diet**				**Peanut meal diet**				**Corn SBM diet**			
			
CP	64.2	58.3	2.0	0.057	78.1	80.3	0.7	0.054	77.5	80.7	1.0	0.048
Indispensable AA												
Arg	82.5	80.6	0.9	0.17	92.9	93.0	0.4	0.88	89.3	89.5	0.6	0.79
His	75.2	65.7	1.4	<0.01	82.5	86.4	1.8	0.15	83.7	84.7	0.6	0.26
Leu	83.9	79.8	0.9	<0.01	88.5	90.4	0.6	0.046	84.7	86.4	0.4	0.022
Ile	71.8	66.7	1.2	0.013	86.3	89.0	0.7	0.015	81.4	84.2	0.6	0.010
Lys	50.6	32.5	1.9	<0.01	74.7	75.5	1.4	0.72	82.2	84.4	1.1	0.19
Met	88.0	86.0	1.0	0.19	88.4	96.9	1.4	<0.01	89.9	90.9	0.9	0.45
Phe	81.5	77.7	0.9	0.011	91.4	92.9	0.5	0.066	84.8	86.6	0.5	0.021
Thr	60.3	54.7	1.5	0.022	75.2	78.5	1.1	0.057	74.0	77.6	1.1	0.040
Trp	49.2	39.5	3.3	0.057	80.2	83.1	0.9	0.054	79.9	82.5	1.0	0.10
Val	68.0	63.5	2.0	0.14	88.5	89.6	0.7	0.24	78.7	81.2	0.9	0.073
Dispensable AA												
Ala	74.7	69.4	1.3	0.013	76.2	80.3	0.8	<0.01	78.4	81.1	1.1	0.12
Asp	65.8	59.8	1.2	<0.01	82.5	84.2	1.4	0.39	79.1	82.4	0.9	0.033
Cys	72.1	60.8	1.7	< 0.01	75.2	78.6	0.9	0.021	73.7	77.2	1.3	0.075
Glu	78.8	74.6	0.8	<0.01	87.7	89.8	1.3	0.28	83.4	86.5	1.2	0.10
Gly	47.6	39.8	3.4	0.13	60.8	61.4	1.9	0.83	67.6	69.7	2.4	0.54
Pro	65.0	63.1	5.1	0.80	73.2	49.6	7.8	0.058	77.1	77.6	3.5	0.92
Ser	71.3	67.3	1.1	0.020	79.0	80.9	0.6	0.034	78.9	82.0	0.8	0.017
Tyr	85.3	80.9	1.0	0.011	85.0	90.3	0.8	<0.01	81.5	82.7	1.5	0.59

SEM, standard error of means; DDGS, distillers dried grains with solubles; SBM, soybean meal.

1) Data of digestibility were mean of six replicates.

2) “−” represents diets without keratinase addition, and “+” represents diets with keratinase addition.

**Table 8:** Standardized ileal digestibility (SID) of crude protein (CP) and amino acids (AA) in 6 experimental diets with or without keratinase addition fed to pigs analyzed within each diet (%)1)

Items	−2)	+2)	SEM	p-value	−	+	SEM	p-value	−	+	SEM	p-value
		
	Rice bran diet				Cottonseed meal diet				Rapeseed meal diet			
CP	52.5	56.8	1.5	0.066	72.2	76.4	1.4	0.056	69.2	71.9	1.3	0.18
Indispensable AA												
Arg	82.3	83.1	0.7	0.46	90.9	92.1	0.8	0.30	82.1	84.4	1.2	0.20
His	70.7	66.4	2.4	0.23	70.9	73.8	3.3	0.55	79.7	75.8	1.0	0.023
Leu	66.0	68.0	1.0	0.19	76.6	80.3	2.0	0.22	80.2	79.5	1.6	0.76
Ile	58.6	61.5	1.3	0.13	75.2	79.3	2.2	0.20	80.0	77.5	1.8	0.42
Lys	62.5	65.8	1.6	0.18	60.9	65.4	2.4	0.21	75.8	76.2	1.2	0.80
Met	55.0	72.4	2.1	<0.01	69.1	71.7	0.8	0.045	89.9	85.0	1.7	0.065
Phe	60.8	65.3	1.6	0.071	84.4	87.0	1.5	0.24	78.3	80.0	1.7	0.48
Thr	52.5	57.4	1.5	0.035	66.0	71.6	2.7	0.17	69.2	70.3	2.2	0.73
Trp	62.3	65.0	1.7	0.28	75.1	79.2	1.3	0.056	71.2	65.9	3.2	0.28
Val	55.3	59.9	1.0	<0.01	75.1	83.0	1.9	0.015	81.0	77.0	2.0	0.18
Dispensable AA												
Ala	59.8	61.9	0.9	0.11	63.9	71.1	2.9	0.10	74.3	74.0	1.3	0.86
Asp	57.4	62.1	1.1	0.012	79.2	82.7	1.9	0.20	70.2	73.9	2.8	0.38
Cys	43.5	53.6	2.9	0.030	72.7	74.0	2.1	0.66	77.8	78.3	1.0	0.71
Glu	70.5	71.4	0.8	0.42	85.1	88.6	1.2	0.068	84.0	85.7	1.8	0.51
Gly	36.3	39.9	3.5	0.48	63.6	64.9	2.9	0.76	63.4	69.7	2.5	0.10
Pro	51.3	53.1	3.6	0.80	54.0	54.3	7.0	0.98	65.7	70.2	7.4	0.67
Ser	55.7	59.4	1.4	0.098	73.5	78.4	2.2	0.15	71.3	73.1	2.3	0.59
Tyr	55.3	65.8	3.3	0.042	79.9	78.6	2.2	0.69	70.9	70.3	1.9	0.83
			
	**Corn DDGS diet**				**Peanut meal diet**				**Corn SBM5 diet**			
			
CP	64.7	58.8	2.0	0.057	78.5	80.8	0.7	0.053	78.0	81.1	1.0	0.048
Indispensable AA												
Arg	82.8	80.9	0.9	0.17	93.0	93.1	0.4	0.90	89.5	89.8	0.6	0.78
His	75.3	65.8	1.4	<0.01	82.6	86.5	1.8	0.15	83.8	84.8	0.6	0.26
Leu	84.1	80.0	0.9	<0.01	88.9	90.7	0.6	0.049	84.9	86.6	0.4	0.022
Ile	72.2	67.1	1.2	0.013	86.6	89.4	0.7	0.017	81.7	84.5	0.6	0.010
Lys	51.3	33.4	1.9	<0.01	75.3	76.0	1.4	0.73	82.5	84.7	1.1	0.19
Met	88.5	86.4	1.0	0.18	89.4	97.7	1.4	<0.01	90.4	91.5	0.9	0.41
Phe	82.0	78.2	0.9	0.012	91.8	93.2	0.5	0.072	85.2	87.0	0.5	0.020
Thr	60.9	55.3	1.5	0.022	75.9	79.1	1.1	0.061	74.5	78.0	1.1	0.040
Trp	50.0	40.3	3.3	0.057	80.7	83.6	0.9	0.058	80.2	82.9	1.0	0.098
Val	68.5	64.0	2.0	0.14	88.9	90.0	0.7	0.27	79.0	81.6	0.9	0.074
Dispensable AA												
Ala	75.0	69.7	1.3	0.013	76.7	80.8	0.8	<0.01	78.8	81.5	1.1	0.12
Asp	66.3	60.3	1.2	<0.01	82.7	84.5	1.4	0.40	79.3	82.6	0.9	0.034
Cys	73.0	61.7	1.7	<0.01	76.4	79.8	0.9	0.021	74.6	78.1	1.3	0.076
Glu	79.0	74.9	0.8	<0.01	87.9	90.0	1.3	0.28	83.6	86.7	1.2	0.097
Gly	48.7	40.8	3.4	0.13	61.4	62.0	1.9	0.84	68.5	70.6	2.4	0.54
Pro	65.5	63.6	5.1	0.80	74.0	50.4	7.8	0.057	77.8	78.2	3.5	0.93
Ser	71.7	67.6	1.1	0.020	79.3	81.2	0.6	0.037	79.2	82.3	0.8	0.017
Tyr	85.7	81.3	1.0	0.012	85.4	90.5	0.8	<0.01	82.0	83.2	1.5	0.59

SEM, standard error of means; DDGS, distillers dried grains with solubles; SBM, soybean meal.

1) Data of digestibility were mean of six replicates.

2) “−” represents diets without keratinase addition, and “+” represents diets with keratinase addition.
